# *ESR1* Gene Variants Are Predictive of Osteoporosis in Female Patients with Crohn’s Disease

**DOI:** 10.3390/jcm8091306

**Published:** 2019-08-24

**Authors:** Iwona Krela-Kaźmierczak, Marzena Skrzypczak-Zielińska, Marta Kaczmarek-Ryś, Michał Michalak, Aleksandra Szymczak-Tomczak, Szymon T. Hryhorowicz, Marlena Szalata, Liliana Łykowska-Szuber, Piotr Eder, Kamila Stawczyk-Eder, Maciej Tomczak, Ryszard Słomski, Agnieszka Dobrowolska

**Affiliations:** 1 Department of Gastroenterology, Human Nutrition and Internal Medicine, Poznan University of Medical Sciences, Przybyszewskiego 49, 60-355 Poznań, Poland; 2 Institute of Human Genetics, Polish Academy of Sciences, Strzeszyńska 32, 60-479 Poznań, Poland; 3 Department of Computer Sciences and Statistics, Poznan University of Medical Sciences, Rokietnicka 7, 60-806 Poznań, Poland; 4 Department of Biochemistry and Biotechnology, Poznan University of Life Sciences, Dojazd 11, 60-637 Poznań, Poland; 5 Department of Psychology, Poznan University of Physical Education, Królowej Jadwigi 27/39, 61-871 Poznań, Poland

**Keywords:** osteoporosis in the course of inflammatory bowel diseases, bone mineral density, *ESR1* gene polymorphism

## Abstract

Decreased bone mass in patients with inflammatory bowel diseases (IBD) is a clinical problem with extremely severe consequences of osteoporotic fractures. Despite its increasing prevalence and the need for mandatory intervention and monitoring, it is often ignored in IBD patients’ care. Determining the biomarkers of susceptibility to bone mineral density disorder in IBD patients appears to be indispensable. We aim to investigate the impact of estrogen receptor gene (*ESR1*) gene polymorphisms on bone mineral density (BMD) in patients with ulcerative colitis (UC) and Crohn’s disease (CD), as they may contribute both, to osteoporosis and inflammatory processes. We characterised 197 patients with IBD (97 with UC, 100 with CD), and 41 controls carrying out vitamin D, calcium and phosphorus serum levels, and bone mineral density assessment at the lumbar spine and the femoral neck by dual-energy X-ray absorptiometry (DXA), *ESR1* genotyping and haplotype analysis. We observed that women with CD showed the lowest bone density parameters, which corresponded to the *ESR1* c.454-397T and c.454-351A allele dose. The *ESR1* gene PvuII and XbaI TA (px) haplotype correlated with decreased femoral neck T-score (OR = 2.75, CI = [1.21–6.27], *P*-value = 0.016) and may be predictive of osteoporosis in female patients with CD.

## 1. Introduction

Patients with inflammatory bowel disease (IBD) frequently present decreased bone mineral density (BMD), which then results in osteopenia and osteoporosis. Depending on the population, osteoporosis occurs in 18–42%, and osteopenia in 22–77% of IBD patients [1,2]. 

Enhanced bone resorption in the course of IBD has been associated with multiple factors, such as chronic active inflammation along the gastrointestinal tract with imbalance in cytokines level, malnutrition with following decreased absorption of calcium and vitamin D, which causes deranged bone mineralisation, or steroid-based therapy that leads to reduced bone formation [1,3]. Nevertheless, epidemiological studies have shown the frequent occurrence of decreased bone mass even in young patients, newly diagnosed with IBD, who were not administered any medication [3,4,5]. The recent meta-analysis performed by Szafors et al. showed that patients with IBD have an increased risk of fractures (17–41% patients), especially in the spine, and significantly decreased BMD at all sites [6]. That generates a largescale problem because osteoporotic fractures cause chronic pain, disability, poor quality of life, and are the leading cause of morbidity [7,8], which advocates research identifying IBD patients burdened with a higher risk of fractures.

Several reports indicate that the factor contributing to decreased bone mass in IBD is, besides inflammatory processes, genetic predisposition [1,9]. In 2000, Schulte et al. showed that genetic variations in the *IL-6* and *IL-1RA* genes correlated with increased bone loss in IBD patients [10]. Then, the GWAS era came through, identifying multiple susceptibility *loci* for low bone mineral density [11]. 

The group of genes potentially contributing to osteoporosis in IBD patients includes the *ESR1* gene (OMIM: 133430) encoding an estrogen receptor alpha (ERα), a ligand-activated transcription factor composed of several domains essential for hormone binding, DNA binding, and activation of transcription [9]. The protein localizes to the nucleus, where it may form a homodimer or a heterodimer with estrogen receptor beta (ERβ). ERα, which is activated by binding with estradiol, is a critical hormone response mediator in estrogen-responsive tissues. The complexity of the receptor with estradiol is mainly responsible for regulating cellular signalling pathways in vivo, and for bone metabolism within the skeletal system [12]. In the latest paper on the topic, Linares et al. confirmed a strong association between low ERβ/ERα ratio and CD clinical and endoscopic activity, while it was not useful in predicting UC activity [13].

Alterations in the *ESR1* gene have been associated with low BMD values; they contribute to increased bone turnover, decreased bone mass, and a higher risk of fractures, both in women and men [14,15]. Numerous studies have investigated sequence variants of the *ESR1* gene in post-menopausal women groups with osteopenia and osteoporosis [16,17,18]. Most of them have focused on two polymorphisms: c.454-397T>C (rs2234693) and c.454-351A>G (rs9340799), localized in intron 1 of the *ESR1* gene, which generates restriction sites for the *Pvu*II and *Xba*I enzymes, respectively [19]. Molvarec et al. concluded that the presence of the c.454-397C allele is associated with increased expression of the *ESR1* gene because it creates a binding site for the B-Myb transcription factor, which can enhance the *ESR1* gene expression or produce different isoforms of ERα, increasing estrogen action [20]. Estrogen inhibits bone turnover by reducing osteoclast-mediated bone resorption and enhancing osteoblast-mediated bone formation [21]. In turn, c.454-351A>G polymorphism has been reported as being associated with fracture risk via mechanisms independent of bone mineral density, such as differences in bone quality, namely bone structure or matrix composition, as well as bone quantity [22,23].

Gonnelli et al. suggested that in post-menopausal women with osteopenia, the primary bone response stressor is the patient’s decreased estrogens status [24]. Recent studies on mice have shown that administering estrogen has a protective effect against IBD and other immune-related diseases [25]. Furthermore, the incidence and progression of IBD differ between sexes [26,27] although, the reported differences are controversial, and the effect of sex hormones on the pathogenesis of IBD remains unclear. Bábíčková and co-authors reported that supplementation with estradiol in ovariectomized mice diminished the severity of colitis; moreover, female mice were partially protected against chemically induced colitis, what might be mediated by estradiol [28].

In recent years, biologic drugs such as TNF inhibitors, integrin receptor antagonists or IL-23 antagonist have gained importance in the treatment of IBD patients [29]. Besides, their potential role in osteoporosis treatment has been proven as well [30,31,32]. Nevertheless, despite high effectiveness, they can have severe side-effects, like infections due to immunosuppression or malignancies. Currently, biosimilars, their equivalent, is available as a more effective, safe and affordable therapeutics [29], making them promising first-line drugs in the therapy of IBD patients, especially those with decreased bone mass. On the other hand, low dose or transdermal estrogens are known to be effective and safe in postmenopausal osteoporosis treatment [33]. In light of the latest research by van der Giessen et al. on IBD patient-derived inflammatory organoid models, estrogen and progesterone function has been proven to decrease pro-inflammatory cytokine production, stimulating wound healing, and increasing barrier function of epithelial cells [34]. This is promising for bone mass maintenance and inflammation alleviation in IBD patients. 

Assuming the existence of genetic susceptibility factors that are shared for IBD and decreased bone mass, we hypothesise that estrogen receptor alteration might influence bone mass loss in IBD patients. In this study, we assess the impact of the *Pvu*II and *Xba*I polymorphisms in the *ESR1* gene on bone mineral density in patients with IBD, with focus on disease entity and sex differences. Besides, we have attempted to determine whether the studied *ESR1* alterations associate with IBD susceptibility. 

## 2. Subjects and Methods

### 2.1. Patients

Patients hospitalized at the Department of Gastroenterology, Human Nutrition and Internal Diseases of Poznan University of Medical Sciences between 2011 and 2014 were prospectively enrolled in the study. The inclusion criteria were as follows: Age between 18 and 50 years for women and 18 and 60 for men, diagnosis of IBD based on cross-sectional imaging and/or endoscopy with a histopathological confirmation. Patients with reduced BMD measured by dual-energy X-ray absorptiometry (DXA) and/or low-energy fractures in clinical history, lack of any other condition that could affect BMD were qualified as IBD with an altered bone mass study group. The exclusion criteria were: Age below 18 years, BMI under 17.5, pregnancy and the coexistence of conditions that could affect bone mineral density (chronic kidney disease, active cancer, liver failure, thyroid disease, rheumatoid arthritis, chronic obstructive pulmonary disease, celiac disease), as well as steroid therapy lasting longer than six months. 

Patients with IBD, and lumbar spine (L1–L4) and femoral neck (FN) T-score over −1, with no fractures in history were qualified as IBD with normal bone mass.

The control group (CG) consisted of healthy volunteers without IBD and with normal bone mass as confirmed by densitometry, reporting no other health problems that may influence the condition of the bone tissue, with no fractures in medical history. 

### 2.2. Ethical Approval

Informed consent was obtained from all individual participants included in the study. The study was approved by the Bioethical Committee of the University of Medical Sciences in Poznan, Poland, under Resolution No. 92/09. All procedures performed in studies involving human participants were in accordance with the ethical standards of the institutional and/or national research committee and the 1964 Helsinki declaration and its later amendments or comparable ethical standards. 

### 2.3. Biochemical Analysis

Serum concentrations of 25-hydroxyvitamin D (25(OH)D) as markers of vitamin D sufficiency in the organism were determined using an electrochemiluminescence binding assay and a Cobas analyser (Roche Molecular Systems, Pleasanton CA, USA). The functional sensitivity of these assays is 4.01 ng/mL (coefficient of variation: 18.5%). Serum calcium and phosphorus levels were determined in all patients (based on reference value ranging from 2.15–2.55 mmol/L for calcium and 0.87–1.45 mmol/L for phosphorus, respectively) and using the Roche assay and Cobas analyzer. The coefficients of variation amounted to 0.8–2.5% and 0.6–0.7% for calcium and phosphorus, respectively.

### 2.4. Measurement of Bone Mineral Density 

Clinical examination-enrolled patients were interviewed with regards to low-energy (osteoporotic) fractures, and a physical examination included the height and weight measurements. Densitometry measurements of the lumbar spine (L1–L4) and femoral neck (FN) were carried out using dual-energy X-ray absorptiometry (DXA) with the Lunar DPX-Plus instrument (GE Healthcare, Madison, WI, USA). Measurements were performed using standard procedures. The apparatus was calibrated daily. The coefficient of variation for DXA of the BMD measurements was 1.25% at the lumbar spine and 1.80% at the femur. The following densitometry parameters were recorded and then taken into account in statistical analyses: Bone mineral density (BMD), Z-score and T-score. Z-score is the difference between the obtained BMD measurements and average BMD matched by age, divided by the standard deviation in the general population. T-score is the difference between the obtained BMD measurements and average BMD for young adults, divided by the standard deviation for young adults.

### 2.5. Molecular Genetic Analysis

Genomic DNA from all subjects was isolated from peripheral blood according to standard procedure, using guanidine isothiocyanate (GTC) and phenol-chloroform extraction followed by precipitation with 96% ethanol. DNA was dissolved in TE buffer and frozen until use. The amplification of the *ESR1* fragment was performed using primers: forward 5′-CTGCCACCCTATCTGTATCTTTTCCTATTCTCC-3′ and reverse: 5′-TCTTTCTCTGCCACCCTGGCGTCGATTATCTG-3′, as was previously described by Kobayashi et al. [19]. A polymerase chain reaction (PCR) was carried out in a volume of 20 μL containing 0.5 U of Taq DNA Polymerase (Sigma Aldrich Co., St. Louis, MO, USA), 2 μL 10 × PCR Buffer (Sigma Aldrich Co., St. Louis, MO, USA), 1.5 mM MgCl_2_, 0.25 mM dNTP (Fermentas GmbH, St. Leon-Rot, Germany), 7.5 pmol of each primer and 100 ng DNA. The amplification was performed using MJ PTC-100 Thermocycler (MJ Research Inc., Waltham, MA, USA). The PCR program started with an initial denaturation at 94 °C for 5 min, followed by 30 cycles of denaturation at 94 °C for 30 s, annealing at 57 °C for 45 s, and extension at 72 °C for 1 min; and a final extension step at 72 °C for 7 min. The PCR products, comprising a part of intron 1 and exon 2 of the *ESR1* gene, were digested with *Pvu*II and *Xba*I restriction enzymes (Fermentas GmbH, St. Leon-Rot, Germany) at 37 °C overnight, producing fragments of 1374 bp (c.454-397C allele) or 936 + 438 bp (c.454-397T allele) and of 1374 bp (c.454-351G) or 981 + 393 bp (c.454-351A), respectively. Electrophoresis in 2% agarose gel stained with ethidium bromide was performed to detect hydrolysis products. Alleles were identified in comparison to control samples determined by Sanger sequencing (Figure S1).

### 2.6. Statistical Analysis

The normality of the distribution and the homogeneity of variable variances were conducted in the experimental groups using the Shapiro-Wilk test and Levene’s test, respectively. In the case of non-concordance with two or at least one condition, the non-parametric Kruskal-Wallis test was used to compare the groups. In the case of statistically significant heterogeneity between groups, multiple comparisons were conducted using Dunn’s test. Evaluation of the association between qualitative variables (the three study groups versus groups carrying different genotypes) were performed using the Chi-squared test. All analyses were conducted using STATISTICA 10.0 software (StatSoft, USA). The frequency differences of particular genotypes and alleles between the groups under study were presented as OR and 95% confidence intervals (CI). The concordance of genotype distributions with the Hardy-Weinberg equilibrium was tested using a calculator on the following website: http://ihg.gsf.de/cgi-bin/hw/hwa1.pl [35]. As indicative of statistical significance, we considered *P*-values below 0.05. 

### 2.7. Linkage Disequilibrium Analysis

The analysis of the linkage disequilibrium for the polymorphisms under study and of the associations between the haplotypes and the clinical data was conducted using the *Haploview v.4.2* software [36].

## 3. Results

### 3.1. Study Group Characteristics

This study included 98 patients with UC (52 females, 46 males), 100 patients with CD (49 females, 51 males) and 41 controls (20 females, 21 males). Mean values for age, height, weight, BMI, lumbar (L2–L4) BMD, femoral neck BMD, T-scores, and Z-scores, were presented according to Becherini et al. [37] and compared between all groups: CD patients, UC patients and controls (Table 1) consistent with earlier reports [38,39,40]. The determined levels of vitamin D, calcium, and phosphorus are compliant with the standards and not have indicated any significant differences between the study groups as previously reported [38]. The comparison of patients with CD and UC has shown relevant differences in body weight and BMI, which were significantly lower in CD patients than in UC patients and healthy controls. Patients with CD also had more significantly lowered L2–L4 lumbar spine BMD and T-score values than patients with UC in comparison with controls. Similarly, the parameters for the femoral neck were noticeably lower in CD patients than in UC, but differences between these patient groups were insignificant (Table 1).

The whole group of women with IBD presented relevantly lower L2-L4 lumbar spine and femoral neck BMD and T-score values than women from the control group (*P*-values < 0.001) (data not shown in tables). Dividing a group of women with IBD due to disease entity, we observed that CD females showed the lowest quantitative bone parameters with significant reduction, even when compared to women with UC. In CD females, we pointed out lower body mass index values than in control females (*P*-value = 0.031). The differences between women with UC and women from the control group were not statistically significant. Men with CD and men with UC had significantly reduced femoral neck BMD (*P*-value = 0.016 and 0.040, respectively), as well as femoral neck T-score (*P*-value = 0.007 and 0.023, respectively) setting against the control group. Interestingly, we noticed decreased BMI measurements in CD men against men with UC and controls (*P*-value = 0.002 and <0.001, respectively). 

Fractures occurred in 26% of the patients with CD and 29.6% of the patients with UC. We did not observe significant differences in fracture proportion between genders (Table 1). 

### 3.2. Analysis of ESR1 Gene Polymorphisms

The studied *Pvu*II (c.454-397T>C) polymorphism in the *ESR1* gene did not present differences in alleles frequency when comparing the whole group of IBD patients and controls (40.7% vs. 39%) or the European population according to the 1000Genomes database. But we have noticed a higher frequency of CC homozygotes in the whole group of IBD patients than in the controls (20.2% vs. 9.8%), however without statistical significance (*P*-value = 0.116). Regarding disease entity, we observed relevantly higher frequency of C allele in UC patients compared to CD patients (OR = 1.68, CI = [1.12–2.52], *P*-value = 0.012). In turn, in CD patients, homozygote TT occurred more frequently than in controls and UC patients (48% vs. 31.7% and 29.6%, respectively). Regarding the c.454-397T as risk allele in the recessive model ([TT] vs. [TC+CC]), the calculated odds ratio was 2.2 (CI = [1.22–3.94], *P*-value = 0.008) (Table 2).

Furthermore, comparing allele frequency in CD patients and general European population according to 1000Genomes database [41] we have observed that c.454-397T allele was overrepresented in CD patients (57.7%; OR = 1.39, CI = [1.01–1.91], *P*-value = 0.043). However, we have made no corrections for the multiple statistical testing. The analysis of *Xba*I alleles and genotypes frequencies in studied groups did not show any statistically significant differences between them (Table 3). 

### 3.3. Correlation of Studied ESR1 Gene Variants with Bone Parameters

Assessing the impact of subjected polymorphisms on bone parameters and taking into account disease entity and sex differences, we observed differences between different *Pvu*II genotypes carriers in the CD women group. We pointed out a noticeable trend: progressively lower values of bone parameters were related to the dose of the c.454-397T allele. Women with TT genotype presented the most decreased all-bone parameters, in particular femoral neck BMD (*P*-value = 0.018), T-score (*P*-value = 0.017) and Z-score (*P*-value = 0.021); however, in post hoc calculations only in the case of femoral neck Z-score, the difference met statistical significance ([TT] vs. [CC]; *P*-value = 0.041) while BMD and T-score were borderline significant ([TT] vs. [CC]; *P*-values were 0.075 and 0.069 respectively). In women with UC, we did not observe such distinct relationships. However, it seems remarkable that in female patients with UC in carriers of CC (*Pvu*II) and GG (*Xba*I) genotypes, BMI values were lower than in carriers of other genotypes (*P*-value = 0.086 and 0.043, respectively, not confirmed in post hoc analyses) (Table 4).

Given the *Xba*I polymorphism, we noticed in women with Crohn’s disease a tendency: lower values of bone parameters were related to the dose of the c.454-351A allele. Depending on the presence of c.454-351A allele, CD female patients presented relevant differences in lumbar spine BMD and T-score (*P*-values were 0.039 and 0.035), although in post hoc analysis, the differences between AA, GA and GG genotype carriers emerged insignificant. 

### 3.4. Haplotypes Analysis and Their Relationship with Bone Parameters

Imputing genotype data to the Haploview v.4.2 software, we observed linkage disequilibrium between the SNPs under study (D’ = 0.987), nevertheless r^2^ coefficient was 0.601, what does not allow determining the allele of one polymorphism from the second one. We observed three possible haplotypes: TA (px), CG (PX) and CA (Px) (with overall frequencies: 54.3%, 33.9% and 11.8% respectively), but noticed relevant differences in haplotypes frequencies between the studied groups. Nevertheless, on performing association analysis, we pointed out in the CD women group, the statistically significant relationship between haplotype and femoral neck T-score; the TA (px) haplotype occurred with higher frequency in patients with decreased femoral neck T-score (T-scores < −1) (OR = 2.75, CI = [1.21–6.27], *P*-value = 0.016). 

## 4. Discussion

Concerning the results of vitamin D, calcium and phosphorus measurements, we assumed that the vast majority of our IBD patients were not malnourished. As far as long-term therapy with steroids is regarded as a bone loss contributing factor, the presented study group also included newly diagnosed patients who had not undergone any treatment yet. These observations have led us to the hypothesis that osteoporosis in IBD patients is not related to low vitamin D or mineral elements’ supply and that seeking of genetic factors is justified. 

The investigated group of patients with Crohn’s disease presented more advanced osteopenia and osteoporosis than patients with ulcerative colitis. We have shown that women with CD had the most decreased bone mineral density parameters. Genetic analysis has revealed that in patients with CD, the c.454-397T allele of the *ESR1* gene is more frequent than in patients with ulcerative colitis, but only slightly overrepresented compared to the controls and European population. In CD women, the c.454-397T and c.454-351A alleles of *ESR1* gene seems to associate with decreased bone mineral density, and TA (px) haplotype occurred related to a lowered femoral neck T-score in this group. 

Low BMD in IBD patients is determined by multiple factors such as chronic inflammation, malnutrition or glucocorticosteroid treatment. Nonetheless, genetic background plays an unquestionable role as well [43]. The impact of steroid treatment on bone mineral density in IBD patients is controversial. Some reported experiments have shown positive correlations between total steroid dose and decreased BMD [44,45], while other studies describe young patients or even children with impaired bone mineral density or osteoporosis, who had not undergone glucocorticosteroid therapy [10,46]. These facts emphasize the role of factors others than treatment in increased bone resorption occurring in IBD patients. Estrogen receptor gene was indicated as one of the genes, potentially determining the bone loss in IBD patients [1,9]. 

The functional role of intron polymorphisms in the *ESR1* gene is still undefined. Molvarec and collaborators pointed out that *ESR1* gene variants can amplify its expression or produce different isoforms, increasing the action of estrogen [20]. Other studies have shown that the absence of the *ESR1* gene in animals causes an exacerbation of the inflammatory process [47], indicating that the action of estrogen is essential in controlling tissue inflammation. In a recent report, Jacenik et al. indicate that estrogen signalling may play a role in local immune response and maintain epithelial homeostasis in a gender- and age-dependent manner, showing dysregulation of estrogen receptors in the intestinal mucosa of IBD patients [48]. 

To the best of our knowledge, there are no previous investigations concerning *ESR1* gene variants in IBD patients. These premises prompted us to undertake the above research. Nevertheless, *ESR1* polymorphisms have been extensively studied in pre- and post-menopausal women. The results of those studies have brought equivocal, and often conflicting, conclusions, depending on the studied population. The impact of *ESR1* polymorphisms on BMD was first assessed in a group of 238 post-menopausal Japanese women, showing that the Px haplotype (presence of alleles c.454-397T and c.454-351A) was predisposed for lower BMD [19]. These results were later corroborated in a study that included a group of 206 British women; it showed a correlation between the Px haplotype and a significantly lower BMD, both at the femoral neck, and the L1–L4 vertebrae [22]. However, further experiments by a Japanese team provided contrary results, suggesting that the PX haplotype correlated with lower BMD [49]. Another report demonstrated that in a group of Chinese men, the pX haplotype was linked to lower trochanter and lumbar spine BMD than non-carriers [50]. Moreover, the px haplotype was correlated with decreased BMD in a group of 634 post-menopausal Dutch women [51]. 

Meanwhile, some analyses found no correlation between *ESR1* variants and BMD or frequency of fractures [31,52,53]. Ioannidis et al. in 2004, performed an *ESR1* gene polymorphisms analysis on a group of 18,917 Caucasian patients from various European regions and reported that *Pvu*II and *Xba*I polymorphisms and their haplotypes did not significantly affect BMD; patients with the XX genotype for *Xba*I had a decreased risk of fracture, independent of BMD [23]. Tang et al. (2013) provided opposite results. They investigated 1838 patients with hip fracture and 14,972 healthy controls and showed that the p allele of *ESR1* might increase the risk of hip fracture, while the *Xba*I polymorphism remained non-associated with fracture risk [53]. These results were contradicted by a 2015 meta-analysis reporting no correlation between the *Pvu*II polymorphism and the risk of fracture in Caucasian and Asian populations [54]. Ignaszak-Szczepaniak et al. studied the *Pvu*II and *Xba*I variants of the *ESR1* gene in the Polish population of premenopausal female patients with Graves’ disease (GD), which also has an autoimmune background. They found that in premenopausal women with GD, homozygous for xx of *Xba*I (AA) and pp of *Pvu*II (TT) had the lowest BMD at the lumbar spine. Moreover, the px haplotype was predisposed to reduced lumbar spine BMD [55]. 

A definite conclusion about the relationship between the studied *ESR1* gene polymorphisms and BMI is problematic because the disease affects BMI in IBD patients. Although the mean BMI did not exceed the lower limit of the norm, in a majority of patients, the condition influenced the value of this index. Nevertheless, we noted that CD females presented lower body mass index values than control females and what is more, CD men showed relevant differences in mean BMI against men with UC and controls. Still, in individuals with CD, we cannot observe a relationship between BMI and the *ESR1* gene haplotypes. Such correlation was slightly apparent in UC woman, exclusively in carriers of homozygous CC (*Pvu*II) and GG (*Xba*I) genotypes (*P*-value = 0.086 and 0.043, respectively). This reinforces the hypothesis that both CD and UC molecular backgrounds are different, and the accompanying osteopenia and osteoporosis has a different underlying substrate. 

This study of *ESR1* variants in IBD patients, including their correlation with BMD values, is pioneering in nature, although it is a challenge to discuss due to the limitation of relatively small studied groups. In the case of IBD patients with osteopenia or osteoporosis, the frequency of these comorbidities restricts a study group size, which should possibly be numerous in association analysis. The calculation power was 80%, but with a 10% probability of type I-error concerning Polish population (38.5 million) and *ESR1* gene rs2234693 (c.454-397T>C) and rs9340799 (c.454-351A>G) polymorphisms alleles frequency (MAF: 0.419 and 0.328, respectively – according to 1000Genomes for European population [41,42]). Another confine of this study is the absence of precise measures of clinical activity of IBD (Crohn’s Disease Activity Index (CDAI) in case of CD and Truelove-Witt’s criteria in case of UC), because the assessment of the real activity of these diseases is challenging and multi-threaded. Nevertheless, all patients enrolled in the study had an active disease, asserted by cross-sectional imaging and/or endoscopy with a histopathological confirmation.

In summary, our results showed that the c.397T allele is more frequent in CD patients and TT homozygote of *PvuII* alteration is more than twice as common as the CC homozygote, compared with UC patients. The different genetic background of CD and UC has already been mentioned [32], and our observations have led us to hypothesize that c.454-397T allele of the *ESR1* gene may associate with Crohn’s disease susceptibility. As c.454-397T allele has correlated with a lower expression of estrogen receptor than c.454-397C allele, it is reasonable to consider this variant as a susceptibility allele. Furthermore, the fact that estrogen treatment inhibits autoimmune inflammatory diseases in animals and decreases pro-inflammatory cytokine production, as well as stimulates wound healing, and increases the barrier function of epithelial cells in IBD patient-derived inflammatory organoid models enhances our hypothesis [28,34,47].

## 5. Conclusions

Our research has revealed that c.454-397T and c.454-351A allele doses associate with decreasing bone density parameters in women with CD (px haplotype), making them potential molecular markers for early identification of patients at increased fracture risk. Moreover, these results are even more impressive in the light of a recent report indicating a relationship between ERβ/ERα ratio and Crohn’s disease activity. Given this data, further studies are needed to understand the mechanism that creates an association between estrogen receptor, increased bone turnover, and inflammation. 

## Figures and Tables

**Table 1:** Characteristics of the investigated groups.

	CD	UC	CG	*P* Value		
*N*	100	98	41	CD vs. UC	CD vs. CG	UC vs. CG
Age (years)	35.59 ± 12.79	39.46 ± 14.69	30.37 ± 8.58	ns.	ns	0.001
Body weight (kg)	63.39 ± 13.71	68.38 ± 14.83	74.63 ± 14.07	0.032	0.001	ns
Height (cm)	171.17 ± 10.19	171.01 ± 9.25	173.05 ± 9.25	ns	ns	ns
BMI (kg/m^2^)	21.51 ± 3.72	23.29 ± 4.28	24.79 ± 3.51	0.004	<0.001	ns
Vit. D (ng/mL)	21.14 ± 11.73	21.74 ± 8.87	21.34 ± 8.94	ns	ns	ns
Ca (mmol/L)	2.32 ± 0.19	2.36 ± 0.14	2.37 ± 0.08	ns	ns	ns
P (mmol/L)	1.13 ± 0.26	1.11 ± 0.28	1.20 ± 0.20	ns	ns	ns
L2–L4 BMD (g/cm^2^)	1.11 ± 0.18	1.16 ± 0.14	1.23 ± 0.08	ns	0.001	0.019
L2–L4 T-score	−0.90 ± 1.45	−0.42 ± 1.15	0.12 ± 0.69	ns	<0.001	0.015
L2–L4 Z-score	−0.12 ± 1.18	−0.12 ± 1.18	0.09 ± 0.64	ns	0.015	ns
Femoral neck BMD (g/cm^2^)	0.94 ± 0.18	0.98 ± 1.18	1.08 ± 1.16	ns	<0.001	0.010
Femoral neck T-score	−0.64 ± 1.30	−0.31 ± 1.22	0.44 ± 1.02	ns	<0.001	0.003
Femoral neck Z-score	−0.25 ± 1.11	0.08 ± 1.06	0.38 ± 0.97	ns	0.006	ns
Patients with bone fractures (n) %	(26) 26%	(29) 29.6%	(0) 0.0%	ns	<0.001	<0.001
**Women**	**CD**	**UC**	**CG**	***P* Value**		
*n*	49	52	20	CD vs. UC	CD vs. CG	UC vs. CG
Women with bone fractures (*n*) %	(13) 26.5%	(15) 28.8%	n.o.	ns	0.011	0.007
BMI (kg/m^2^)	21.17 ± 4.04	22.10 ± 4.32	23.39 ± 3.09	ns	0.031	ns
L2–L4 BMD (g/cm^2^)	1.06 ± 0.17	1.17 ± 0.13	1.21 ± 0.08	0.001	0.005	ns
L2–L4 T score	−1.17 ± 1.41	−0.34 ± 1.15	0.06 ± 0.65	0.002	0.004	ns
L2–L4 Z score	−0.54 ± 1.26	0.13 ± 1.17	0.21 ± 0.65	0.020	0.056	ns
Femoral neck BMD (g/cm^2^)	0.85 ± 0.15	0.93 ± 1.14	0.99 ± 0.09	0.014	0.004	ns
Femoral neck T-score	−1.08 ± 1.27	−0.49 ± 1.11	0.08 ± 0.72	0.033	<0.001	ns
Femoral neck Z-score	−0.50 ± 1.13	0.03 ± 1.00	0.20 ± 0.70	0.064	0.015	ns
**Men**	**CD**	**UC**	**CG**	***P* Value**		
*n*	51	47	21	CD vs. UC	CD vs. CG	UC vs. CG
Men with bone fractures (*n*) %	(13) 25.5%	(14) 29.8%	n.o.	ns	0.013	0.006
BMI (kg/m^2^)	21.58 ± 3.35	24.85 ± 3.83	25.27 ± 5.98	0.002	<0.001	ns
L2–L4 BMD (g/cm^2^)	1.20 ± 0.18	1.18 ± 0.14	1.27 ± 0.08	ns	ns	ns
L2–L4 T score	−0.64 ± 1.45	−0.51 ± 1.16	0.17 ± 0.74	ns	0.089	ns
L2–L4 Z score	−0.39 ± 1.34	−0.42 ± 1.13	−0.27 ± 0.63	ns	ns	ns
Femoral neck BMD (g/cm^2^)	1.03 ±0.16	1.05 ± 0.16	1.17 ± 0.16	ns	0.016	0.040
Femoral neck T-score	−0.23 ± 1.19	−0.11 ± 1.31	0.78 ± 1.16	ns	0.007	0.023
Femoral neck Z-score	−0.01 ± 1.05	0.21 ± 1.14	0.56 ± 1.17	ns	ns	ns

Abbreviations: IBD—inflammatory bowel disease, UC—ulcerative colitis, CD—Crohn’s disease, BMD—bone mineral density, CG—control group. Quantitative data were presented as mean with standard deviation. n.o.—not observed, ns—non-significant (only statistically significant or borderline significant results were shown).

**Table 2:** Alleles and genotypes frequencies for *ESR1* c.454-397T>C (*Pvu*II) polymorphism.

	*ESR1* c.397T>C (*Pvu*II)				
	Genotype Frequencies (%)			Allele Frequencies (%)	
	TT	TC	CC	T * 1000Genomes: 57.7%	C * 1000Genomes: 42.3%
IBD (all patients) *n* = 198	77 (38.9%)	81 (41.0%)	40 (20.2%)	235 (59.3%)	161 (40.7%)
UC patients (*n* = 98)	29 (29.6%)	46 (46.9%)	23 (23.5%)	104 (53.1%)	92 (46.9%)
CD patients ** (*n* = 100)	48 (48.0%)	35 (35.0%)	17 (17.0%)	131 (65.5%)	69 (34.5%)
CG (*n* = 41)	13 (31.7%)	24 (58.5%)	4 (9.8%)	50 (61.0%)	32 (39.0%)
Comparisons of allelic and genotypic frequencies between groups under study					
	[TT + TC] vs. [CC]	[TT] vs. [TC + CC]	[TT] vs. [CC]	[T] vs. [C]	[C] vs. [T]
CG vs. IBD OR, 95% CI *P*-value	OR = 0.43 [0.14–1.27] *P* = 0.116	OR = 0.73 [0.36–1.50] *P* = 0.388	OR = 1.69 [0.517–5.516] *P* = 0.382	OR = 1.07 [0.66–1.74]	OR = 0.93 [0.57–1.52]
				*P* = 0.784	
CG vs. UC OR, 95% CI *P*-value	OR = 0.35 [0.11–1.09] *P* = 0.062	OR = 1.11 [0.50–2.43] *P* = 0.804	OR = 2.58 [0.74–8.97] *P* = 0.129	OR = 1.38 [0.82–2.34]	OR = 0.72 [0.43–1.22]
				*P* = 0.226	
CG vs. CD OR, 95% CI *P*-value	OR = 0.58 [0.17–1.68] *P* = 0.273	**OR = 0.50** **[** **0.13–0.70]** ***P*** ** = 0.004**	OR = 1.15 [0.33–4.02] *P* = 0.825	OR = 0.82 [0.48–1.40]	OR = 1.22 [0.72–2.10]
				*P* = 0.472	
CD vs. UC OR, 95% CI *P*-value	OR = 0.67 [0.33–1.35] *P* = 0.257	**OR = 2.20** **[** **1.22–3.94]** ***P*** ** = 0.008**	**OR = 2.24** **[** **1.03–4.88]** ***P*** ** = 0.041**	**OR = 1.68** **[1.12–2.52]**	**OR = 0.60** **[** **0.40–0.89]**
				***P*** ** = 0.012**	

In bold were marked statistically significant results (*P*-value < 0.05). No corrections for the multiple statistical testing were made. CG—control group, [TT+TC] vs. [CC]—dominant model, risk allele: T, [TT] vs. [TC+CC]—recessive model, risk allele: C, * Allele frequencies for European population according to 1000 Genomes Project have been gathered from https://www.ncbi.nlm.nih.gov/projects/SNP/snp_ss.cgi?ss=ss1322898669 website [41]. ** HWE analysis revealed discordance in CD patients group.

**Table 3:** Alleles and genotypes frequencies for *ESR1* c.454-351A>G (*Xba*I) polymorphism.

		*ESR1* c.454-351A>G (*Xba*I)				
		Genotype Frequencies (%)			Allele Frequencies (%)	
		GG	GA	AA	G * 1000Genomes: 30.8%	A * 1000Genomes: 69.2%
IBD (all patients) *n* = 198		21 (9.6%)	98 (49.0%)	79 (41.4%)	140 (35.4%)	256 (64.6%)
UC patients (*n* = 98)		11 (11.2%)	47 (48.0%)	40 (40.8%)	69 (35.2%)	127 (64.8%)
CD patients (*n* = 100)		10 (10.0%)	51 (51.0%)	39 (39.0%)	71 (35.5%)	129 (64.5%)
CG (*n* = 41)		2 (4.9%)	19 (46.3%)	20 (48.8%)	23 (28.0%)	59 (72.0%)
Comparisons of allelic and genotypic frequencies between groups under study						
	[GG + GA] vs. [AA]		[GG] vs. [GA + AA]	[GG] vs. [AA]	[G] vs. [A]	[A] vs. [G]
CG vs. IBD OR, 95% CI *P*-value	OR = 0.70 CI = [0.36–1.37] *P* = 0.293		OR = 2.31 CI = [0.52–10.28] *P* = 0.258	OR = 2.66 CI = [0.58–12.29] *P* = 0.196	OR = 1.40 CI = [0.83–2.37]	OR 0.71 CI = [0.42–1.20]
					*P* = 0.204	
CG vs. UC OR, 95% CI *P*-value	OR = 0.72 CI = [0.35–1.51] *P* = 0.387		OR = 2.47 CI = [0.52–11.65] *P* = 0.241	OR = 2.75 CI = [0.56–13.61] p = 0.201	OR = 1.39 CI = [0.79–2.45]	OR = 0.71 CI = [0.41–1.26]
					*P* = 0.248	
CG vs. CD OR, 95% CI *P*-value	OR = 0.67 CI = [0.32–1.40] *P* = 0.285		OR = 2.17 CI = [0.45–10.35] *P* = 0.322	OR = 2.56 CI = [0.51–12.84] *P* = 0.239	OR = 1.412 CI = [0.81–2.48]	OR = 0.71 CI = [0.40–1.24]
					*P* = 0.228	
CD vs. UC OR, 95% CI *P*-value	OR = 0.93 CI = [0.53–1.64] *P* = 0.794		OR = 0.88 C.I. = [0.36–2.17] *P* = 0.780	OR = 0.932 CI = [0.36–2.44] *P* = 0.887	OR = 1.01 CI = [0.67–1.53]	OR = 0.99 CI = [0.65–1.49]
					*P* = 0.951	

No corrections for the multiple statistical testing were made. CG—control group, [GG + GA] vs. [AA]—dominant model, risk allele: G, [GG] vs. [GA + AA]—recessive model, risk allele: A, * Allele frequencies for European population according to 1000Genomes Project have been gathered from https://www.ncbi.nlm.nih.gov/projects/SNP/snp_ss.cgi?ss=ss1322898670 website [42].

**Table 4:** Bone parameters in correlation with *ESR1* gene *Pvu*II (c.454-397T>C) and *Xba*I (c.454-351A>G) genotypes.

**Females** ***Pvu*II**	**CD (*n* = 49)** **Average** ** ± SD**				*P*-Value *	**UC (*n* = 52)** **Average** **± SD**			*P*-Value *
	**CC** ***n* = 8 (16.3%)**	**TC** ***n* = 24 (49.0%)**		**TT** ***n* = 17 (34.7%)**		**CC** ***n* = 12 (23.1%)**	**TC** ***n* = 25 (48.1%)**	**TT** ***n* = 15 (28.8)**	
BMI (kg/m^2^)	20.95 ± 3.66	22.25 ± 4.68		19.46 ± 2.52	0.128	20.49 ± 4.43	21.82 ± 3.82	23.85 ± 4.67	0.086
BMD L2–L4 (g/cm^2^)	1.13 ± 0.20	1.09 ± 0.17		1.00 ± 0.15	0.127	1.10 ± 0.14	1.18 ± 0.15	1.19 ± 0.16	0.330
L2–L4 T-score	−0.62 ± 1.70	−0.96 ± 1.40		−1.69 ± 1.21	0.133	−0.80 ± 1.14	−0.31 ± 1.11	−0.12 ± 1.31	0.389
L2–L4 Z-score	−0.23 ± 1.32	−0.34 ± 1.38		−0.93 ± 1.03	0.272	−0.14 ± 1.18	0.21 ± 1.08	0.08 ± 1.32	0.697
Femoral neck BMD	0.94 ± 0.15	0.88 ± 0.16		0.77 ± 0.11	**0.018**	0.87 ± 0.13	0.94 ± 0.13	0.95 ± 0.14	0.269
Femoral neck T-score	−0.37 ± 1.27	−0.83 ± 1.33		−1.73 ± 0.93	**0.017**	−0.94 ± 1.18	−0.36 ± 1.09	−0.31 ± 1.02	0.274
Femoral neck Z-s	0.07 ± 1.04	−0.23 ± 1.14		−1.09 ± 0.91	0.021 **	−0.30 ± 0.93	0.12 ± 1.09	−0.05 ± 0.90	0.541
**Males** ***Pvu*II**	**CD (*n* = 51)** **Average** **± SD**				*P*-Value *	**UC (*n* = 46)** **Average** **± SD**			*P*-Value *
	**CC** ***n* = 10 (19.6%)**	**TC** ***n* = 35 (68.6%)**		**TT** ***n* = 6 (11.8%)**		**CC** ***n* = 11 (23.9%)**	**TC** ***n* = 21 (45.7%)**	**TT** ***n* = 14 (30.4%)**	
BMI (kg/m^2^)	21.28 ± 2.31	21.84 ± 3.66		21.26 ± 3.36	0.694	24.56 ±4.78	25.23 ± 4.19	24.48 ± 2.43	0.819
BMD L2–L4 (g/cm^2^)	1.11 ± 0.11	1.13 ± 0.19		1.28 ± 0.17	0.127	1.14 ± 0.12	1.18 ± 0.12	1.18 ± 0.16	0.636
L2–L4 T-score	−0.98 ± 0.69	−0.82 ± 1.52		0.40 ± 1.45	0.125	−0.80 ± 1.00	−0.44 ± 1.10	−0.34 ± 1.36	0.653
L2–L4 Z-score	−0.66 ± 0.60	−0.58 ± 1.43		0.62 ± 1.29	0.108	−0.73 ± 0.80	−0.30 ± 1.15	−0.39 ± 1.40	0.408
Femoral neck BMD	1.02 ± 0.07	1.00 ± 0.16		1.14 ± 0.18	0.134	1.05 ± 0.20	1.04 ± 0.12	1.04 ± 0.17	0.993
Femoral neck T-score	−0.33 ± 0.55	−0.42 ± 1.24		0.64 ± 1.29	0.123	−0.13 ± 1.50	−0.22 ± 0.94	0.03 ± 1.56	0.909
Femoral neck Z-score	−0.05 ± 0.56	−0.22 ± 1.05		0.87 ± 1.22	0.101	0.18 ± 1.24	0.11 ± 0.77	0.31 ± 1.48	0.810
**Females** ***Xba*I**	**CD (*n* = 49)** **Average** **± SD**				*P*-Value *	**UC (*n* = 52)** **Average** **± SD**			*P*-Value *
	**GG** ***n* = 3 (6.1%)**	**GA** ***n* = 24 (49.0%)**	**AA** ***n* = 22 (44.9%)**			**GG** ***n* = 6 (11.5%)**	**GA** ***n* = 25 (48.1%)**	**AA** ***n* = 21 (40.4%)**	
BMI (kg/m^2^)	22.09 ± 6.05	21.11 ± 3.49	20.89 ± 4.47		0.758	19.88 ± 2.78	21.19 ± 4.00	23.83 ± 4.55	**0.043**
BMD L2–L4 (g/cm^2^)	1.26 ± 0.15	1.08 ± 0.17	1.01 ± 0.16		**0.039**	1.12 ± 0.06	1.16 ± 0.17	1.18 ± 0.15	0.754
L2–L4 T-score	0.53 ± 1.24	−0.98 ± 1.40	−1.58 ± 1.31		**0.035**	−0.64 ± 0.54	−0.44 ± 1.25	−0.20 ± 1.22	0.721
L2–L4 Z-score	0.44 ± 1.11	−0.30 ± 1.31	−0.91 ± 1.15		0.104	0.12 ± 0.52	0.17 ± 1.25	0.02 ± 1.19	0.660
Femoral neck BMD	0.97 ± 0.09	0.88 ± 0.16	0.81 ± 0.14		0.152	0.85 ± 0.09	0.93 ± 0.15	0.95 ± 0.13	0.309
Femoral neck T-score	−0.12 ± 0.74	−0.85 ± 1.37	−1.43 ± 1.14		0.127	−1.16 ± 0.93	−0.40 ± 1.23	−0.38 ± 0.94	0.312
Femoral neck Z-score	−0.04 ± 0.35	−0.21 ± 1.15	−0.83 ± 1.11		0.148	−0.29 ± 0.45	0.10 ± 1.23	−0.11 ± 0.79	0.733
**Males** ***Xba*I**	**CD (*n* = 51)** **Average** **± SD**				*P*-Value *	**UC (*n* = 46)** **Average** **± SD**			*P*-Value *
	**GG** ***n* = 7 (13.7%)**	**GA** ***n* = 27 (53.0%)**		**AA** ***n* = 17 (33.3%)**		**GG** ***n* = 5 (10.9%)**	**GA** ***n* = 22 (47.8%)**	**AA** ***n* = 19 (41.3%)**	
BMI (kg/m^2^)	20.91 ± 2.70	21.66 ± 3.64		21.97 ± 3.27	0.582	24.74 ± 7.17	25.13 ± 3.64	24.54 ± 3.10	0.834
BMD L2–L4 [g/cm^2^]	1.15 ± 0.10	1.12 ± 0.20		1.18 ± 0.18	0.663	1.07 ± 0.13	1.17 ± 0.12	1.19 ± 0.14	0.190
L2–L4 T-score	−0.85 ± 0.77	−0.88 ± 1.62		−0.37 ± 1.32	0.504	−1.20 ± 1.20	−0.57 ± 1.10	−0.23 ± 1.17	0.326
L2–L4 Z-score	−0.58 ± 0.56	−0.58 ± 1.53		−0.19 ± 1.23	0.632	−1.15 ± 0.64	−0.45 ± 1.10	−0.22 ± 1.26	0.230
Femoral neck BMD	1.04 ± 0.05	0.98 ± 0.16		1.08 ± 0.15	0.110	0.98 ± 0.23	1.03 ± 0.13	1.07 ± 0.16	0.484
Femoral neck T-score	−0.22 ± 0.36	−0.56 ± 1.27		0.15 ± 1.17	0.145	−0.58 ± 1.75	−0.26 ± 1.06	0.15 ± 1.36	0.678
Femoral neck Z-score	0.07 ± 0.28	−0.28 ± 1.08		0.25 ± 1.11	0.276	−0.30 ± 1.44	0.06 ± 0.94	0.45 ± 1.21	0.509

In bold are marked statistically significant results (*P*-value < 0.05). No corrections for the multiple statistical testing were made. * *P*-value for multiple comparisons, ** Post hoc analysis *P*-value = 0.041 for [CC]vs[TT].

## Supplementary Materials

The following are available online at https://www.mdpi.com/2077-0383/8/9/1306/s1, Figure S1: Genotyping of *ESR1* gene SNPs by PCR-RFLP and Sanger sequencing. **a.** Genotyping of polymorphism rs2234693 (c.454-397T>C) in the *ESR1* gene. PCR products have been digested with *Pvu*II restriction enzyme and separated in agarose gel. Lanes: 1, 3, 10, 13 demonstrate CC homozygous genotype (undigested product size: 1374 bp); lanes: 2, 5, 8, 9, 12 demonstrate TC heterozygous genotype (digestion products size: 1374, 936 and 438 bp); lanes: 4, 6, 7, 11 demonstrate TT homozygous genotype (digestion products size: 936 and 438 bp). M—size marker pUC/*Taq*I+*Pvu*II. At the bottom of the figure, confirmation of results by Sanger sequencing with reverse primer. The polymorphic site is highlighted. **b.** Genotyping of polymorphism rs9340799 (c.454-351A>G) in the *ESR1* gene. PCR products have been digested with *Xba*I restriction enzyme and separated in agarose gel. Lanes: 2, 9, 12 demonstrate GG homozygous genotype (undigested product size: 1374 bp); lanes: 1, 3, 5, 7, 13 demonstrate AG heterozygous genotype (digestion products size: 1374, 981 and 393 bp); lanes: 4, 6, 8, 10 and 11 demonstrate AA homozygous genotype (digestion products size: 981 and 393 bp). M—size marker λ/*Eco*RI+*Hind*III. At the bottom of the figure, confirmation of results by Sanger sequencing with reverse primer. The polymorphic site is highlighted.
