# Supplementary figures and images for: ESR1 Gene Variants Are Predictive of Osteoporosis in Female Patients with Crohn’s Disease
Source: J Clin Med. 2019 Aug 24;8(9):1306. doi: 10.3390/jcm8091306 (PMC6780775; doi:10.3390/jcm8091306)

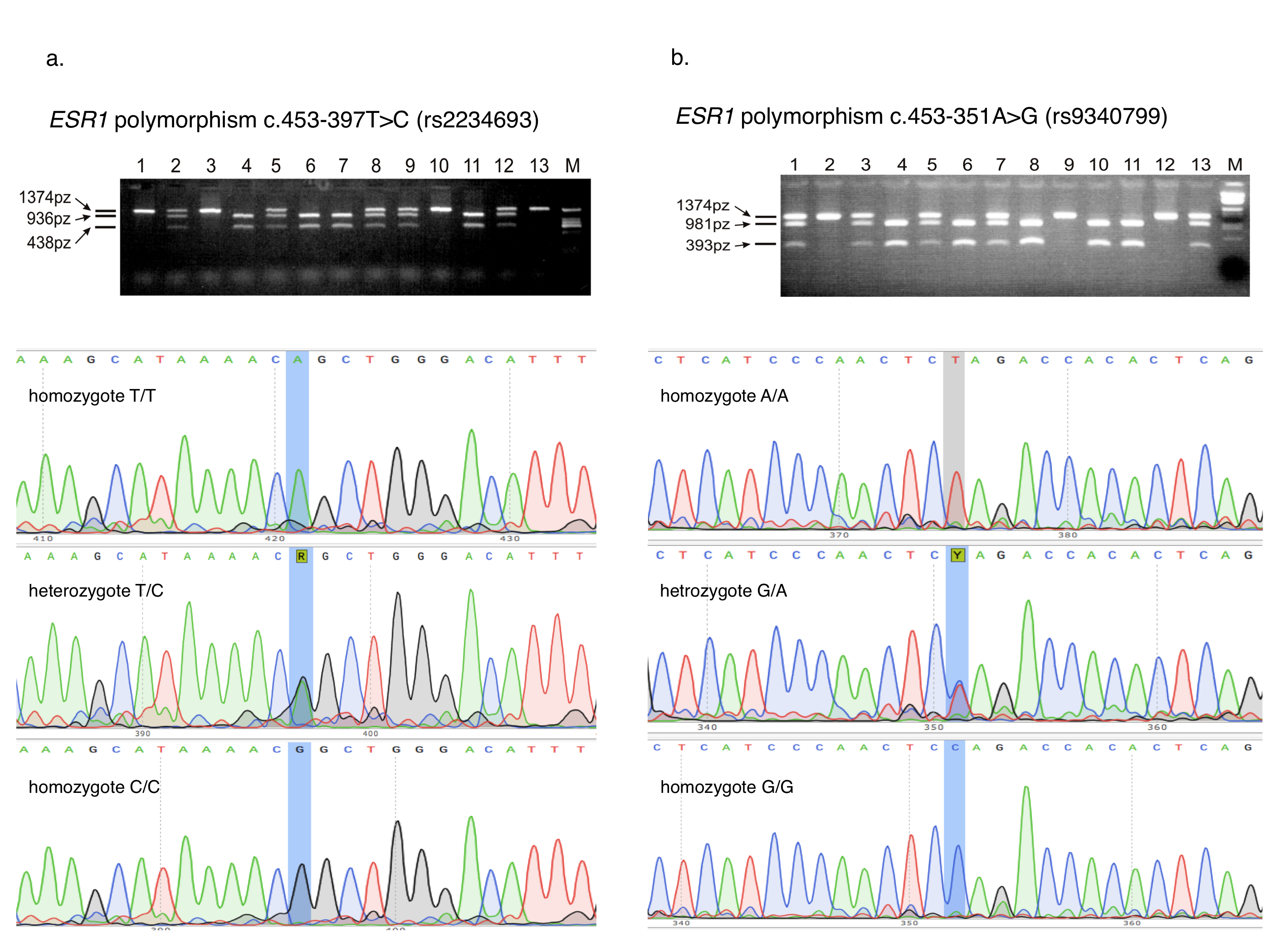

Supplement: Supplementary file 1 [file jcm-08-01306-s001.zip › jcm-565911-supplementary.tif]
